# FGF12 is a novel component of the nucleolar NOLC1/TCOF1 ribosome biogenesis complex

**DOI:** 10.1186/s12964-022-01000-4

**Published:** 2022-11-21

**Authors:** Martyna Sochacka, Radoslaw Karelus, Lukasz Opalinski, Daniel Krowarsch, Martyna Biadun, Jacek Otlewski, Malgorzata Zakrzewska

**Affiliations:** 1 Department of Protein Engineering, Faculty of Biotechnology, University of Wroclaw, Joliot-Curie 14a, 50-383 Wrocław, Poland; 2 Department of Protein Biotechnology, Faculty of Biotechnology, University of Wroclaw, Joliot-Curie 14a, 50-383 Wrocław, Poland

**Keywords:** FGF12, Protein-protein interaction, Nucleolus, Ribosome biogenesis, NOLC1, TCOF1

## Abstract

Among the FGF proteins, the least characterized superfamily is the group of fibroblast growth factor homologous factors (FHFs). To date, the main role of FHFs has been primarily seen in the modulation of voltage-gated ion channels, but a full picture of the function of FHFs inside the cell is far from complete. In the present study, we focused on identifying novel FGF12 binding partners to indicate its intracellular functions. Among the identified proteins, a significant number were nuclear proteins, especially RNA-binding proteins involved in translational processes, such as ribosomal processing and modification. We have demonstrated that FGF12 is localized to the nucleolus, where it interacts with NOLC1 and TCOF1, proteins involved in the assembly of functional ribosomes. Interactions with both NOLC1 and TCOF1 are unique to FGF12, as other FHF proteins only bind to TCOF1. The formation of nucleolar FGF12 complexes with NOLC1 and TCOF1 is phosphorylation-dependent and requires the C-terminal region of FGF12. Surprisingly, NOLC1 and TCOF1 are unable to interact with each other in the absence of FGF12. Taken together, our data link FHF proteins to nucleoli for the first time and suggest a novel and unexpected role for FGF12 in ribosome biogenesis.

**Video Abstract**

**Supplementary Information:**

The online version contains supplementary material available at 10.1186/s12964-022-01000-4.

## Background

The fibroblast growth factor (FGF) family includes 22 genes found in humans that share sequence and structural similarity [1, 2]. FGFs bind to their specific fibroblast growth factor receptors (FGFRs) and stimulate intracellular signaling pathways [3]. FGFs-induced cellular responses are critical for developmental processes and their deregulation is associated with cancer and metabolic diseases [4]. FGFs fall into three major groups: canonical FGFs (comprising subfamilies 1, 4, 7, 8 and 9), endocrine FGFs, referred to as the FGF19 subfamily, and fibroblast growth factor homologous factors (FHFs), until recently considered intracellular FGFs, termed the FGF11 subfamily [2, 5].

The FHF subfamily consists of four proteins: FGF11, FGF12, FGF13 and FGF14. They contain a 120–130 amino acid conserved domain that folds into a β-trefoil structure, homologous to other FGFs [6]. FHFs lack an N-terminal signal sequence [7, 8] and their secretion, even in an unconventional manner, has not yet been documented. Moreover, FHF proteins are unable to stimulate proliferation in FGFR-positive cells [9]. They were therefore considered to be exclusively intracellular proteins whose function is independent of cell surface FGFRs. This view has recently been partially challenged by us and others, who have shown that recombinant FHFs are capable of directly binding and activating FGFR and inducing receptor-downstream signaling pathways [9, 10], leading to an anti-apoptotic cellular response [9]. Each intracellular FHF is represented by at least two distinct isoforms, differing at the N-terminus and resulting from alternative splicing [8]. The isoform determines the cellular localization of FHFs, with the “a” isoform containing an N-terminal bipartite nuclear localization signal (NLS) directing the protein to the nucleus, while the shorter “b” isoform, lacking the NLS, is predominantly localized in the cytosol [11].

The main function of FHF proteins, so far, has been attributed to their cytosolic localization, where they act as intracellular modulators of plasma membrane voltage-gated ion channels [11–16]. In addition, cytosolic FHFs form signal transducing complexes with partner proteins such as islet-brain-2 (IB2), NF-κB essential modulator (NEMO), hypoxia-inducible factor-1a (HIF-1a), casein kinase 2 (CK2), glycogen synthase kinase 3 (GSK3) and (JAK2) [1, 17–25]. Although FHFs have been observed in the cell nucleus [26], their biological role there remains a mystery.

Based on the first comprehensive mass spectrometry (MS)-based analysis of the FGF12 interactome, we present here the newly identified FGF12 interactors. We found a group of nucleolar proteins, including NOLC1 (nucleolar and coiled-body phosphoprotein 1; Nopp140) and TCOF1 (treacle ribosome biogenesis factor 1; Treacle), that provide insights into a new and unexpected role for FGF12 in the nucleus.

## Methods

### Antibodies and reagents

The anti-FGF12 antibody (#PA5-67182) was from Thermo Fisher Scientific (Waltham, MA, USA). The primary antibodies: anti-NOLC1 (#sc-374033), anti-TCOF1 (#sc-374536), anti-THRAP3 (#sc-133250), anti-BCLAF1 (#sc-101388), anti-dyskerin (#sc-373956), anti-FGF12 (#sc-81947), anti-SBP (#sc-101595), anti-histone H3 (#sc-10809), anti-His-tag (#sc-8036) were from Santa Cruz (Dallas, TX, USA). The primary antibodies: anti-phospho-FGFR (Tyr653/Tyr654) (p-FGFR) (#06-1433), anti-phospho-p44/42 (Thr202/Tyr204) MAP kinase (p-ERK1/2) (#9101), anti-p44/42 MAP kinase (ERK1/2) (#9102) were from Cell Signaling (Danvers, MA, USA). The primary antibodies: anti-NOLC1 (#HPA037366) and anti-γ-tubulin (tubulin) (#T6557) were from Sigma-Aldrich (St Louis, MO, USA). The anti-GFP antibody (#50430-2-AP) was from ProteinTech (Chicago, IL, USA). Horseradish peroxidase-conjugated secondary antibodies and the anti-mouse secondary antibody conjugated to Alexa Fluor 594 (#715-585-150) were from Jackson ImmunoResearch (Cambridge, UK). The secondary anti-rabbit antibody conjugated to Alexa Fluor 594 (#A11037) was from Thermo Fisher Scientific.

Pierce Anti c-myc Magnetic Beads (#88843) and Streptavidin agarose resin (#20353) were from Thermo Fisher Scientific. Ni Sepharose 6 Fast Flow resin (#GE17-5318-02) was from GE Healthcare (Chicago, IL, USA). siRNA against NOLC1 (#sc-38127) and TCOF1 (#sc-61707) were from Santa Cruz. The non-targeting control siRNA (#D-001810-01-50) and siRNA against FGF12 [27] was ordered from Horizon Discovery (Waterbeach, UK). Calf intestinal alkaline phosphatase (CIP) was from Sigma-Aldrich (#P4978-1KU). Nuclease was from Thermo Fisher Scientific (#88701).

### Cells

The human osteosarcoma (U2OS) cell line was obtained from American Type Culture Collection (ATCC, Manassas, VA, USA). Cells were cultured in Dulbecco’s Modified Eagle’s Medium (DMEM) (Biowest, Nauille, France) supplemented with 10% fetal bovine serum (FBS) (Thermo Fisher Scientific) and antibiotics (100 U/ml penicillin, 100 µg/ml streptomycin) (Thermo Fisher Scientific). Stable U2OS-FGF11-GFP-myc, U2OS-FGF12-GFP-myc, U2OS-FGF13-GFP-myc, U2OS-FGF14-GFP-myc and U2OS-FGF12-SBP cell lines were obtained from U2OS cells transfected with pcDNA3.1 vectors containing sequences encoding the 'a' isoforms of individual FHFs in fusion with GFP-myc or SBP (Gene Universal, Newark, DE, USA) using FuGene HD transfection reagent (Roche, Indianapolis, IN, USA). Cells were replated 24 h after transfection and cultured in selection medium (growth medium with 1 mg/ml geneticin (G-418) (BioShop, Canada) until colony formation was observed. Colonies were transferred using cloning discs (Sigma-Aldrich) to 6-well plates and then to T-75 cm^2^ flask for continued culture under the same conditions. Expression of FGFs was confirmed by western blotting using antibodies specific for GFP and FGFs. Human embryonic kidney cells (HEK 293) obtained from ATCC were cultured in DMEM (Biowest) supplemented with 10% fetal bovine serum and antibiotics (100 U/ml penicillin, 100 µg/ml streptomycin). Mouse embryo fibroblast cells (NIH3T3) were obtained from ATCC and were cultured in DMEM supplemented with 10% bovine serum (BS) and antibiotics (100 U/ml penicillin, 100 µg/ml streptomycin). All cell lines were grown in a 5% CO_2_ atmosphere at 37 °C. Cells were seeded onto tissue culture plates one day before the experiments.

### Recombinant proteins

Phusion Inverse PCR mutagenesis was applied to obtain the 131–181 FGF12 deletion mutant (FGF12Δ-His), using pDEST17-FGF12 as a template. The primers contained a STOP codon at the protein truncation site. Expression and purification of recombinant his-tagged FGF12 variants were performed as previously described [9].

### siRNA transfection

siRNA transfections were performed with DharmaFECT Transfection Reagents (Horizon, Cambridge, UK) according to the manufacturer’s instructions. Cells were transfected with 100 nM siRNA against NOLC1, TCOF1 or FGF12, and after 24 h the transfection medium was replaced with complete medium. Control cells were transfected with 100 nM non-targeting siRNA. Cells were incubated in a 5% CO_2_ atmosphere at 37 °C for another 48 h.

### Fluorescence microscopy

To investigate the localization of FGFs proteins stably expressed in fusion with GFP, U2OS-FGF11-mGFP-myc, U2OS-FGF12-mGFP-myc, U2OS-FGF13-mGFP-myc, U2OS-FGF14-mGFP-myc cells were fixed with 4% paraformaldehyde. Nuclei were stained with NucBlue Live (Thermo Fisher Scientific). For quantification, the mean fluorescence intensity of each compartment was measured using Zeiss ZEN 2.3 software. To calculate the amount of fluorescent FHFs in each compartment, the mean intensity of fluorescence was multiplied by the area of the respective compartment.

To analyze the co-localization of FGF12-mGFP-myc and their protein partners, U2OS-FGF12-mGFP-myc cells were fixed with 4% paraformaldehyde and permeabilized with 0.1% Triton in PBS. Cells were then stained with mouse anti-NOLC1, anti-TCOF1, anti-dyskerin, anti-THRAP3, anti-BCLAF1 primary antibodies and Alexa Fluor 594-conjugated anti-mouse secondary antibody. Cell nuclei were stained with NucBlue Live dye. Wide-field fluorescence microscopy was carried out using a Zeiss Axio Observer Z1 fluorescence microscope (Zeiss, Oberkochen, Germany). Images were captured using an LD-Plan-Neofluar 40 × /0.6 Korr M27 objective and an Axiocam 503 camera. The red PLA signal was visualized using a 540/552 nm bandpass excitation filter and a 575/640 nm bandpass emission filter, and the FGF12-mGFP-myc signal was visualized with a 450/490 nm bandpass excitation filter and a 500/550 nm bandpass emission filter. The NucBlue Live signal was visualized using a 335/383 nm bandpass excitation filter and a 420/470 nm bandpass emission filter. Images were processed with Zeiss ZEN 2.3 software.

### Proximity ligation assay (PLA)

To analyze the interaction of FGF12, NOLC1 and TCOF1, U2OS-FGF12-GFP-myc cells or HEK 293 cells were fixed with 4% paraformaldehyde and permeabilized with 0.1% Triton in PBS. Cells were then stained with antibodies against NOLC1 and FGF12, TCOF1 and FGF12 or NOLC1 and TCOF1 in combination with secondary PLA probes according to the manufacturer’s protocols (Duolink In Situ PLA, Sigma-Aldrich).

### Pull-down

To confirm the interaction of FGF12 with proteins identified in MS experiments, recombinant FGF12 was bound to Ni Sepharose Resin (1 h, 4 °C) and incubated with U2OS cell lysed in lysis buffer (0.15 M KCl, 40 mM Tris, 1% NP-40, 1 mM EDTA, 0.1 mM PMSF, pH 7.2), supplemented with protease and phosphatase inhibitor cocktails (Roche), overnight at 4 °C. The resin was washed three times with PBS and bound proteins were eluted with Laemmli sample buffer. Proteins were separated by SDS-PAGE and analyzed by Western blotting using the following antibodies: anti-NOLC1, anti-TCOF1, anti-BCLAF1, anti-THRAP3 and anti-FGF12.

To study the interaction between FHFs and NOLC1 or TCOF1, U2OS-FGF12-GFP-myc (or U2OS-FGF12-SBP), U2OS-FGF11-mGFP-myc, U2OS-FGF12-mGFP-myc, U2OS-FGF13-mGFP-myc, U2OS-FGF14-mGFP-myc cells were lysed in lysis buffer (0.15 M KCl, 40 mM Tris, 1% NP-40, 1 mM EDTA, 0.1 mM PMSF, pH 7.2) supplemented with protease and phosphatase inhibitor cocktails and incubated with Pierce Anti c-myc Magnetic Beads or Streptavidin agarose. The resin was washed two times with PBS and the bound proteins were eluted with Laemmli sample buffer. Proteins were separated by SDS-PAGE and analyzed by Western blotting using antibodies recognizing: GFP, NOLC1 or TCOF1. U2OS cells were used as a control.

### 2D Blue native/SDS-PAGE

U2OS-FGF12-SBP cell was lysed in lysis buffer (0.15 M KCl, 40 mM Tris, 1% NP-40, 1 mM EDTA, 0.1 mM PMSF, pH 7.2) supplemented with protease and phosphatase inhibitor cocktails and incubated with Streptavidin agarose resin overnight in 4 °C. The resin was then washed three times with PBS, and bound proteins were eluted with 4 mM biotin in lysis buffer and separated by BN-PAGE on 4–13% gradient gels. For two-dimensional analysis, individual complexes were analyzed in the 1st dimension by BN-PAGE, and then isolated and separated by SDS-PAGE in the 2nd dimension. After electrophoresis, proteins were transferred to a PVDF membrane and detected with anti-NOLC1, anti-TCOF1 and anti-FGF12 antibodies.

### Cell fractionation

To fractionate cells into cytoplasmic and nuclear fractions, U2OS cells stably expressing FGF11-mGFP-myc, FGF12-mGFP-myc, FGF13-mGFP-myc, FGF14-mGFP-myc were lysed in lysis buffer (0.15 M KCl, 40 mM Tris, 0.1% NP-40, 1 mM EDTA, 0.1 mM PMSF, pH 7.2) supplemented with protease and phosphatase inhibitor cocktails (Roche). The soluble fraction was designated as the cytoplasmic fraction. The insoluble fraction, obtained by centrifugation of the lysates followed by sonication, was designated as the nuclear fraction. FGFs present in different fractions were concentrated by binding to Pierce Anti c-myc Magnetic Beads. The presence of FGF11, FGF12, FGF13, FGF14 or marker proteins (ERK1/2 and histone-H3), confirming the purity of the fractions, was analyzed by immunoblotting.

To isolate nucleoli, U2OS-FGF12a-GFP-myc cells were lysed in lysis buffer for 20 min on ice. After centrifugation, the pellet was resuspended in 0.5 M sucrose and sonicated, layered over a cushion of 1 M sucrose and centrifuged to pellet nucleoli. The nucleoli were washed by resuspension in 0.5 M sucrose followed by centrifugation. The nucleoli were resuspended in the same volume of Laemmli sample buffer used to prepare sample from total lysate. The presence of FGF12 and NOLC1 was analyzed by immunoblotting.

### FGFR1 activation and downstream signaling

Serum-starved NIH3T3 cells were treated with FGF12Δ-His or FGF1 (as control) in the presence of heparin (10 U/ml) (Sigma-Aldrich) for 45 min. Cells were lysed with Laemmli sample buffer and lysates were subjected to SDS-PAGE and western blotting.

### Mass spectrometry analysis

To identify binding partners of FGF12, we used recombinant His-tagged FGF12, which was coupled to Ni-NTA resin and incubated with U2OS cell lysate. Mass spectrometry analysis was performed by the Mass Spectrometry Laboratory at the Institute of Biochemistry and Biophysics, Polish Academy of Sciences (Warsaw, Poland). Beads with proteins were subjected to a standard trypsin digestion procedure, where proteins were reduced with 5 mM TCEP for 1 h at 60 °C, blocked with 10 mM MMTS for 10 min at RT and digested overnight with trypsin (0.1 mg/ml). After digestion, the peptides were dried in a SpeedVac and purified using a modified SP3 procedure [28]. Briefly, tryptic peptides were resuspended in 20 µl of water with 0.1% formic acid (FA) and bound in the presence of acetonitrile (ACN) to 15 µl of bead mix prepared by combining equal parts of Sera-Mag Carboxyl hydrophilic and hydrophobic particles (09-981-121 and 09-981-123, GE Healthcare). Peptides were washed twice with 1 ml of ACN and eluted with 200 mM ammonium hydroxide. The dried peptides were then resuspended in 50 µl of 2% ACN and 0.1% FA. The peptide mixtures were applied in equal volumes of 20 µl to an RP-18 pre-column (Waters, Milford, MA, USA) using water containing 0.1% FA as a mobile phase and then transferred to an RP-18 nano-HPLC column (internal diameter 75 µM, Waters) using an ACN gradient (0–35% ACN in 160 min) in the presence of 0.1% FA at a flow rate of 250 nl/min. The column outlet was coupled directly to the ion source of the Q Exactive mass spectrometer (Thermo Fisher Scientific) operating in DDA mode with Top12 ions subjected to the fragmentation after each full scan with a scan range from 300 to 1650 m/z. Each analysis was preceded by a blank run to ensure no cross-contamination from previous samples.

Acquired MS/MS data from triplicates were preprocessed with Mascot Distiller software (v. 2.6, MatrixScience) and searched using Mascot Search Engine (MatrixScience, Mascot Server 2.6) against the human proteins deposited in Swissprot 2019_11 database (20,442 sequences). To reduce mass errors, peptide and fragment mass tolerance settings were determined separately for each LC-MS/MS runs after recalibration of the measured mass, as described previously [29]. Statistical assessment of peptide assignment confidence was based on a target/decoy database search strategy [25]. This procedure provided q-value estimates for each peptide spectrum match in the data set. All queries with q-values > 0.01 were removed from further analysis. The mass calibration and data filtering described above were performed using MScan software developed in-house (http://proteom.ibb.waw.pl/mscan/).

## Results

### FGF12 is a novel component of nucleolar protein complexes

**Fig. 1:**
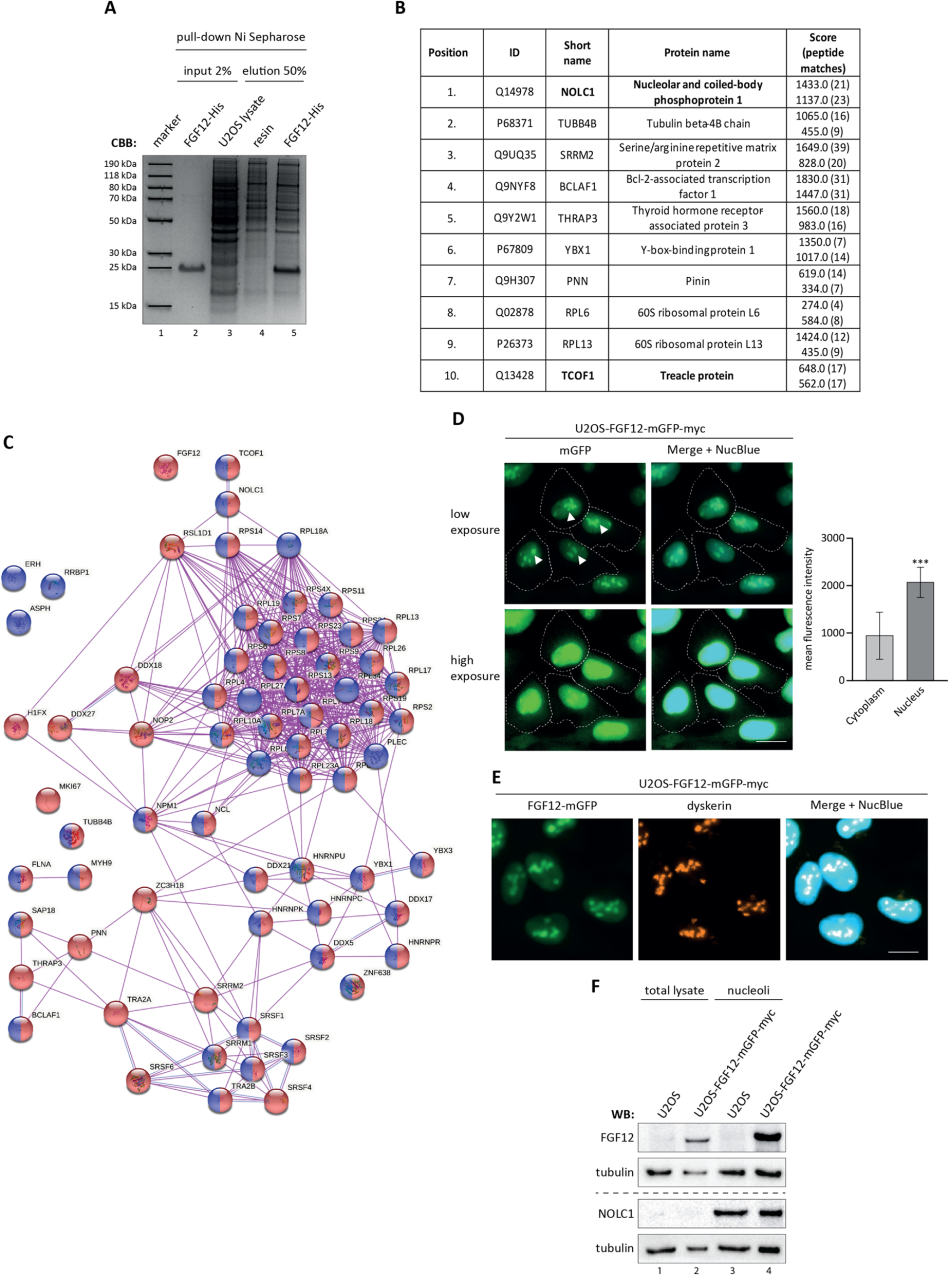
Identification of FGF12 partner proteins. **A** Recombinant FGF12-His was bound to Ni Sepharose and incubated with U2OS cell lysate. Proteins were eluted with Laemmli sample buffer, separated with SDS-PAGE and stained with CBB. **B** Top 10 results of identifying proteins by MS that specifically bound to FGF12-His in pull-down experiments. ID is the Uniprot protein identifier, the abbreviated name is also the gene name. The score and peptide match for each of the two repeats of the experiment are given. **C** FGF12 interaction network identified by affinity purification followed by mass spectrometry. Proteins were classified using STRING database (http://www.string-db.org/). Blue spheres represent nuclear proteins and red spheres represent cytosolic proteins. The lines connecting the spheres represent the experimentally confirmed interactions. **D** Cellular localization of FGF12. U2OS cells stably expressing FGF12-mGFP-myc (U2OS-FGF12-mGFP-myc) were fixed, then cell nuclei were stained with NucBlue Live, and analyzed with fluorescence microscopy. The dashed line indicates the cell area and the arrows indicate the nucleoli. The scale bar represents 20 μm. The graph shows the mean fluorescence intensity in the nucleus and cytoplasm. Data presented are means ± SD of 20 cells. A paired student’s t-test was used for statistical analysis; ***p < 0.001. **E** Co-localization of FGF12-mGFP-myc with the nucleolar marker, dyskerin. U2OS-FGF12-mGFP-myc cells were fixed, permeabilized and stained with a primary antibody against dyskerin and Alexa Fluor 594-conjugated anti-mouse secondary antibody. Cell nuclei were labeled with NucBlue Live and cells were analyzed by fluorescence microscopy. The scale bar represents 20 μm. **F** U2OS-FGF12-mGFP-myc cells were lysed, and nucleoli were isolated by sonication and centrifugation in buffer with increasing sucrose concentration. The presence of FGF12 and NOLC1 in nucleoli was analyzed by immunoblotting. An antibody against γ-tubulin was used to ensure equal loading of the samples.

FGF12 is a pleiotropic protein involved in modulation of voltage-gated ion channel, cellular signaling, reactive oxygen species (ROS) and apoptosis [9, 30–35]. The versatility of FGF12 activities implicates that this protein may be part of various protein complexes inside the cell. However, unbiased screening to decipher the map of FGF12 intracellular interactions has not yet been performed. We used functional recombinant His-tagged FGF12 [9], which was coupled to Ni Sepharose resin and incubated with U2OS cell lysate (Fig. 1A). Proteins specifically bound to FGF12-His were identified by MS, yielding more than 70 proteins (Additional file 1: Table S1). Among the top hits, several ribosomal proteins and two major components of the nucleolar ribosome biogenesis complex were found: nucleolar and coiled-body phosphoprotein 1 (NOLC1; Nopp140; Uniprot ID: Q14978) and treacle ribosome biogenesis factor 1 (TCOF1; Treacle; Uniprot ID: Q13428) (Fig. 1B, C). Interestingly, functional classification of all identified FGF12 interactors indicated multiple ribosomal components closely related to NOLC1/TCOF1 *via* RSL1D1, RPS14 and RPL18A (Fig. 1C).

In the next step, we verified the localization of FGF12 in U2OS cells. To this end, we stably transfected U2OS cells with the FGF12-mGFP-myc expression construct (Additional file 2: Fig. S1) and monitored the subcellular localization of FGF12-mGFP-myc with fluorescence microscopy. We observed a weak mGFP signal in the cytoplasm, while most of the fluorescence was detected in the nuclei of U2OS cells (Fig. 1D). Surprisingly, inside the nuclei, the FGF12-mGFP-myc signal accumulated in characteristic, well-defined structures specific to the nucleoli (Fig. 1D), compartments rich in the NOLC1/TCOF1 complex and several other proteins identified by us in MS experiments (Additional file 1: Table S1). The nucleolar localization of FGF12 was validated by fluorescence microscopy using an antibody against a specific nucleolar protein, dyskerin (Fig. 1E) [36–38]. Next, we isolated nucleoli from U2OS cells stably transfected with FGF12-mGFP-myc and confirmed the presence of FGF12 in the NOLC1-positive nucleoli-enriched fraction (Fig. 1F).

**Fig. 2:**
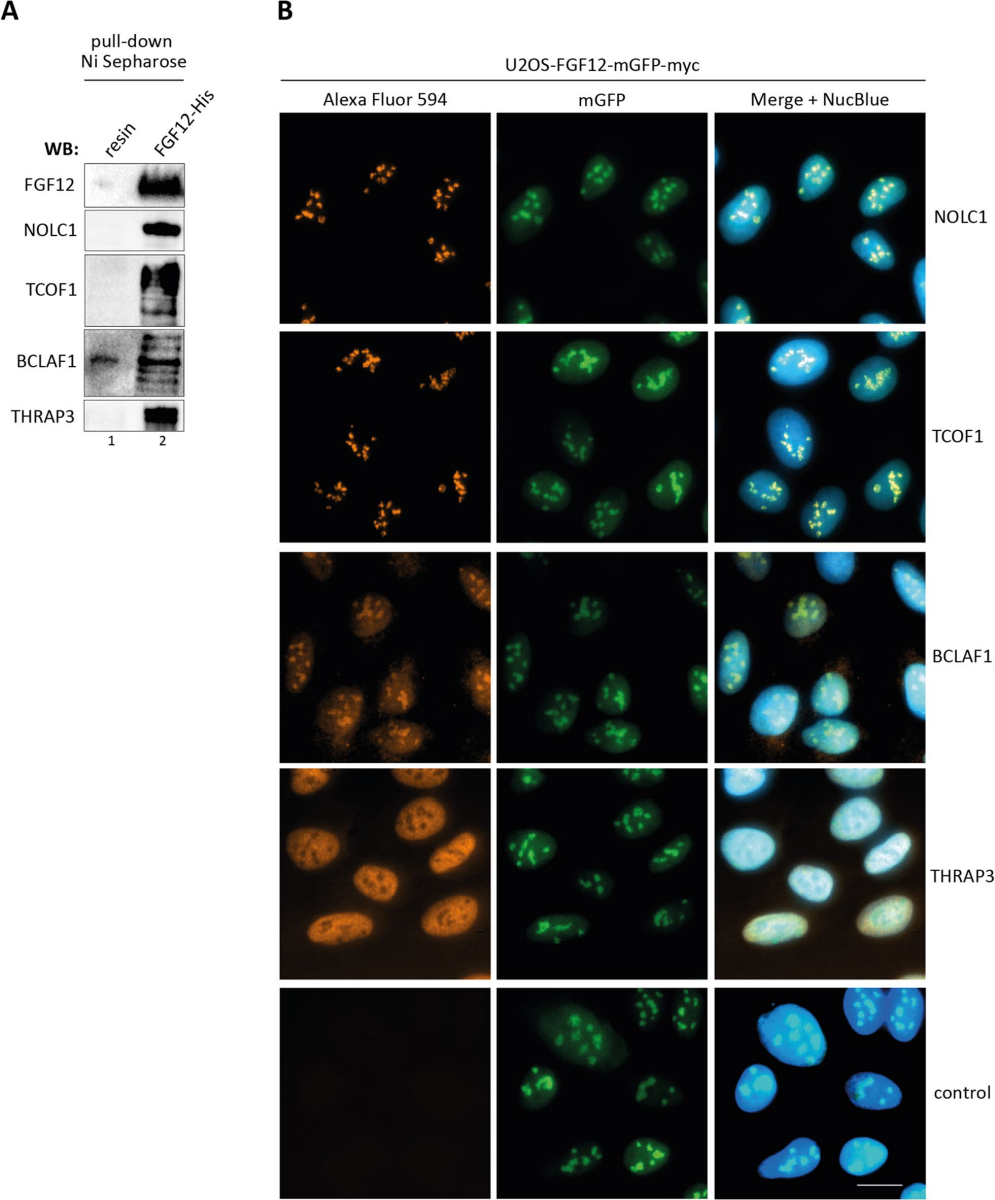
Interaction of FGF12 with nucleolar proteins. **A** Verification of the results of MS experiments. Recombinant FGF12-His was bound to Ni Sepharose and incubated with U2OS cells lysate. FGF12-bound proteins were eluted and analyzed by wester blotting using specific antibodies. **B** Co-localization of FGF12-mGFP-myc with identified partner protein. To analyze the co-localization of FGF12-mGFP-myc with NOLC1, TCOF1, BCLAF1, THRAP3, U2OS-FGF12-mGFP-myc cells were fixed, permeabilized and stained with appropriate primary antibodies and Alexa Fluor 594-conjugated anti-mouse secondary antibody. Cell nuclei were labeled with NucBlue Live and cells were analyzed by fluorescence microscopy. The scale bar represents 20 μm.

To analyze whether the nucleolar localization of FGF12 is unique to FGF12 or whether it is a common feature shared by the family of fibroblast growth factor homologous factors (FHFs), we stably transfected U2OS cells with expression vectors providing FGF11-mGFP-myc, FGF13-mGFP-myc and FGF14-mGFP-myc production (Additional file 2: Fig. S1A) and proceeded with fluorescence microscopy. As shown in Additional file 2: Fig. S1B and S1C, all FHF members were found in the nucleoli of U2OS cells, albeit in different amounts. While the FGF12-mGFP-myc and FGF13-mGFP-myc signal predominated in nuclei, with accumulation in nucleoli, the FGF11-mGFP-myc was detected in both nuclei and cytoplasm, with only trace fluorescence in nucleoli (Additional file 2: Fig. S1B, C). For the FGF14-mGFP-myc protein, a signal was still visible in the nucleoli, but it was weaker than that observed for FGF12-mGFP-myc and FGF13-mGFP-myc. For all FHFs, we confirmed cytoplasmic and nuclear localization using cell fractionation and western blotting (Additional file 2: Fig. S1D).We verified MS interaction data using Ni Sepharose pull-down with recombinant FGF12-His and cell lysate prepared from U2OS cells. Western blotting with antibodies specific for the top MS-listed nucleolar proteins: NOLC1, TCOF1, BCLAF1 and THRAP3 revealed their efficient-co-purification with FGF12-His (Fig. 2A). Using immunolabeling and fluorescence microscopy, we confirmed co-localization of FGF12-mGFP-myc with NOLC1, TCOF1, BCLAF1 in cell nucleoli and THRAP3 in the nucleus of U2OS cells (Fig. 2B). These data indicate that FGF12 is a novel nucleolar protein involved in a multifaceted interaction network centered on the NOLC1/TCOF1 complex involved in the binding of multiple ribosomal proteins (Fig. 1C).

### FGF12 interacts with NOLC1/TCOF1 in a phosphorylation-dependent manner

**Fig. 3:**
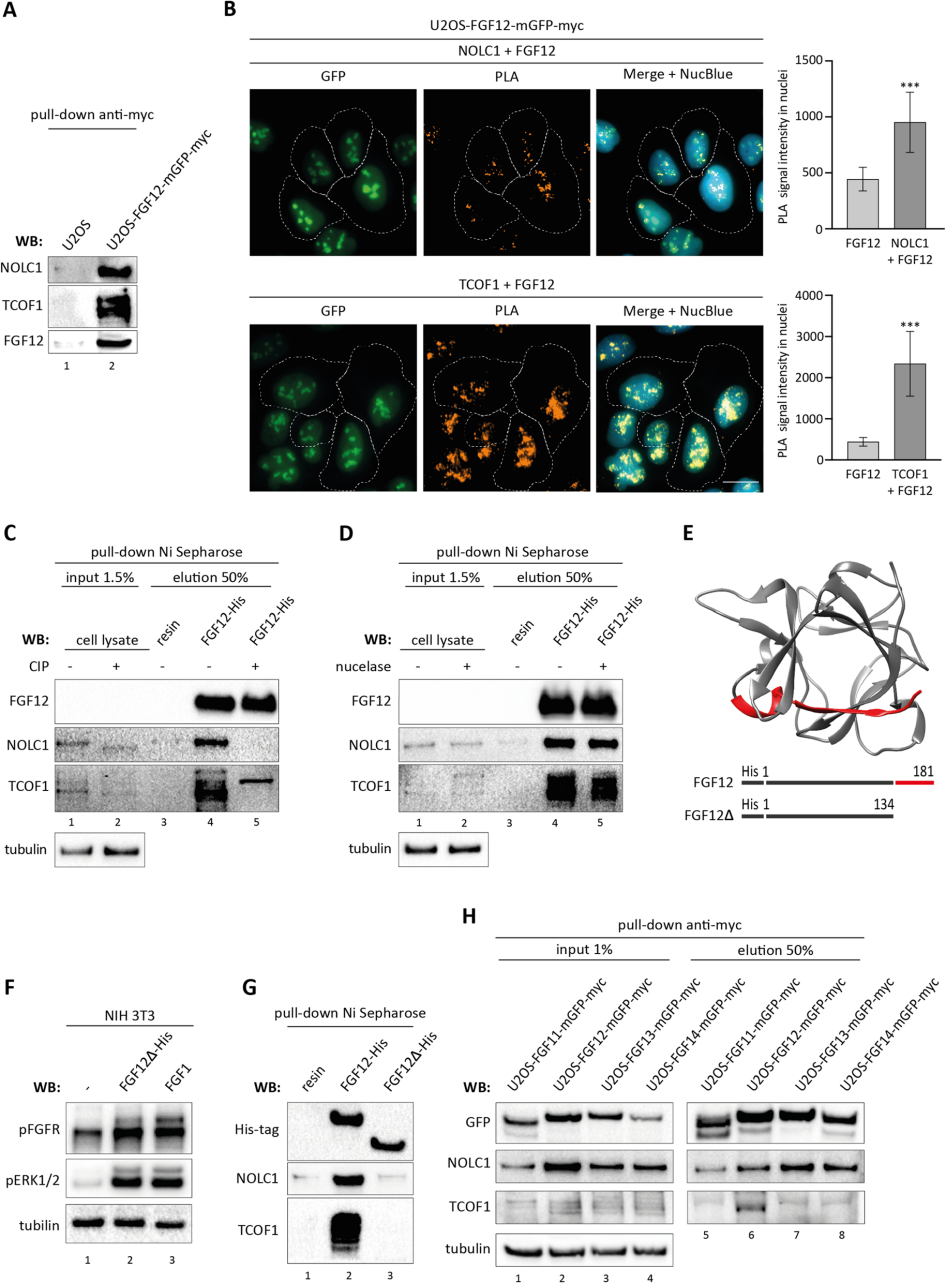
Characterization of FGF12 complexes with NOLC1 and TCOF1. **A** U2OS-FGF12-mGFP-myc and U2OS (control) cells were lysed and the co-purification of NOLC1 and TCOF1 with FGF12-mGFP-myc was determined with SDS-PAGE and western blotting. **B** In situ proximity ligation assay (PLA) using rabbit anti-FGF12, mouse anti-NOLC1 or anti-TCOF1 antibodies in U2OS-FGF12-mGFP-myc cells. Cell nuclei were labeled with NucBlue Live and cells were analyzed by fluorescence microscopy. The dashed line indicates the cell area. The scale bar represents 20 μm. Data shown in the graphs are mean PLA signal intensities in nuclei ± SD from three independent experiments (100 cells in total). Student’s t-test was applied for statistical analysis; ***p < 0.001. **C** Pull-down experiment with recombinant FGF12-His bound to Ni Sepharose and U2OS cell lysate incubated with calf intestinal alkaline phosphatase (CIP), analyzed by SDS-PAGE and western blotting using anti-FGF12, anti-NOLC1 or anti-TCOF1 antibodies. An antibody against γ-tubulin was used to ensure equal loading of input samples. **D** Pull-down experiment with recombinant FGF12-His bound to Ni Sepharose and nuclease-treated U2OS cell lysate, analyzed by SDS-PAGE and western blotting using anti-FGF12, anti-NOLC1 or anti-TCOF1 antibodies. An antibody against γ-tubulin was used to ensure equal loading of input samples. **E** Structural (based on PDB 1Q1U structure) and bar representation of FGF12 with the deleted region in FGF12Δ-His marked in red (upper panel). **F** Biological activity of FGF12Δ-His variant. Serum-starved NIH 3T3 cells were incubated with recombinant FGF12Δ-His (~ 1 µg/ml) or FGF1 (control, ~ 0.1 µg/ml) for 45 min, and activation of cell signaling was assessed by western blotting with antibodies specific for activated FGFR and ERK1/2 (pFGFR; pERK1/2). An antibody against γ-tubulin was used to ensure equal loading. **G** Interaction of FGF12Δ-His mutant with NOLC1 and TCOF1. Recombinant FGF12-His and FGF12Δ-His were bound to Ni Sepharose and incubated with U2OS cells lysate. Elution fractions were subjected to western blot analysis with anti-NOLC1, anti-TCOF1 and anti-His-tag antibodies. **H **Lysates of U2OS cells expressing FGF11-mGFP-myc, FGF12-mGFP-myc, FGF13-mGFP-myc, FGF14-mGFP-myc were incubated with anti-myc agarose. Co-purification of NOLC1 and TCOF1 with FHFs was determined with western blotting using anti-GFP, anti-NOLC1 and anti-TCOF1 antibodies. An antibody against γ-tubulin was used to ensure equal loading of input samples.

Because the NOLC1/TCOF1 complex was at the top of the MS hit list for FGF12 binding partners and because of the critical role of NOLC1/TCOF1 in ribosome biogenesis, we decided to focus on the FGF12-NOLC1/TCOF1 interaction. To confirm that FGF12 and NOLC1/TCOF1 form complex(es) inside cells, we applied pull-down experiments with anti-myc agarose using U2OS and U2OS cells stably transfected with FGF12-mGFP-myc. As shown by SDS-PAGE followed by western blotting analysis in Fig. 3A, both NOLC1 and TCOF1 were efficiently co-purified with FGF12-mGFP-myc. We confirmed these data by streptavidin-agarose pull-down using U2OS cells stably transfected with FGF12-SBP (Additional file 3: Fig. S2A, B). In addition, we used streptavidin-agarose pull-down from U2OS stably expressing FGF12-SBP cells (U2OS-FGF12-SBP) to purify FGF12-SBP complexes under native conditions (eluted with biotin) and analyzed the presence of NOLC1 and TCOF1 in FGF12 complexes using 2D-PAGE. As shown in Additional file 3: Fig. S2C, the FGF12-SBP signals overlapped significantly with those of NOLC1 and TCOF1, indicating efficient purification of high molecular weight FGF12/NOLC1/TCOF1 complexes.

Furthermore, we employed a proximity ligation assay (PLA) to study the subcellular localization of FGF12 complexes with NOLC1 and TCOF1. In U2OS cells stably expressing FGF12-mGFP-myc, PLA signals for both FGF12-NOLC1 and FGF12-TCOF1 pairs largely overlapped with mGFP fluorescence, indicating that FGF12/NOLC1/TCOF1 complexes are mainly localized to nucleoli (Fig. 3B, Additional file 4: Fig. S3). Using a similar experimental setup, we also confirmed the interaction between FGF12, NOLC1 and TCOF1 in HEK293 cells lacking ectopic FGF12 (Additional file 5: Fig. S4).

NOLC1 and TCOF1 are heavily phosphorylated nucleolar proteins. To investigate whether the interaction of FGF12 with NOLC1 and TCOF1 depends on their phosphorylation status, we used a Ni Sepharose pull-down assay with recombinant FGF12-His and calf intestinal alkaline phosphatase (CIP)-treated U2OS cell lysates. CIP efficiently de-phosphorylated both NOLC1 and TCOF1, which is evident as an acceleration of their migration in the gel (Fig. 3C, lanes 1, 2). De-phosphorylation completely inhibited the interaction of NOLC1 with FGF12 and significantly blocked the binding of TCOF1 to FGF12 (Fig. 3C, lane 5). In contrast, the interaction of FGF12 with NOLC1 and TCOF1 is nucleic acid-independent, as nuclease treatment had no effect on the co-purification efficiency of FGF12 with NOLC1 and TCOF1 (Fig. 3D, lane 5).

To investigate which region of FGF12 is involved in NOLC1 and TCOF1 recognition, we prepared a recombinant FGF12Δ-His variant containing most of the FGF core domain but lacking 47 C-terminal residues, including the 12th β-strand (Fig. 3E). FGF12Δ-His retained the ability to bind and activate FGFR1 (Fig. 3F, lane 2), but was virtually incapable of binding NOLC1 and TCOF1 (Fig. 3G, lane 3).

Since we have identified, not only FGF12, but all other FHFs: FGF11, FGF13 and FGF14 in nucleoli, we decided to test whether these proteins are capable of binding NOLC1/TCOF1. To this end, U2OS cells stably transfected with FGF11-mGFP-myc, FGF12-mGFP-myc, FGF13-mGFP-myc, FGF14-mGFP-myc were subjected to anti-myc agarose pull-down, and the co-purification efficiency of NOLC1 and TCOF1 was assessed by western blotting. While NOLC1 was co-purified with all FHF members, TCOF1 was only detected in the elution fraction of FGF12-mGFP-myc cells (Fig. 3H).

All these data strongly indicate that the nucleolar pool of FGF12 is part of the NOLC1/TCOF1 complex. The association of FGF12 with NOLC1/TCOF1 is strictly dependent on NOLC1 and TCOF1 phosphorylation and requires the C-terminal region of FGF12.

### Complexes of NOLC1 and TCOF1 required FGF12 for their nucleolar localization

**Fig. 4:**
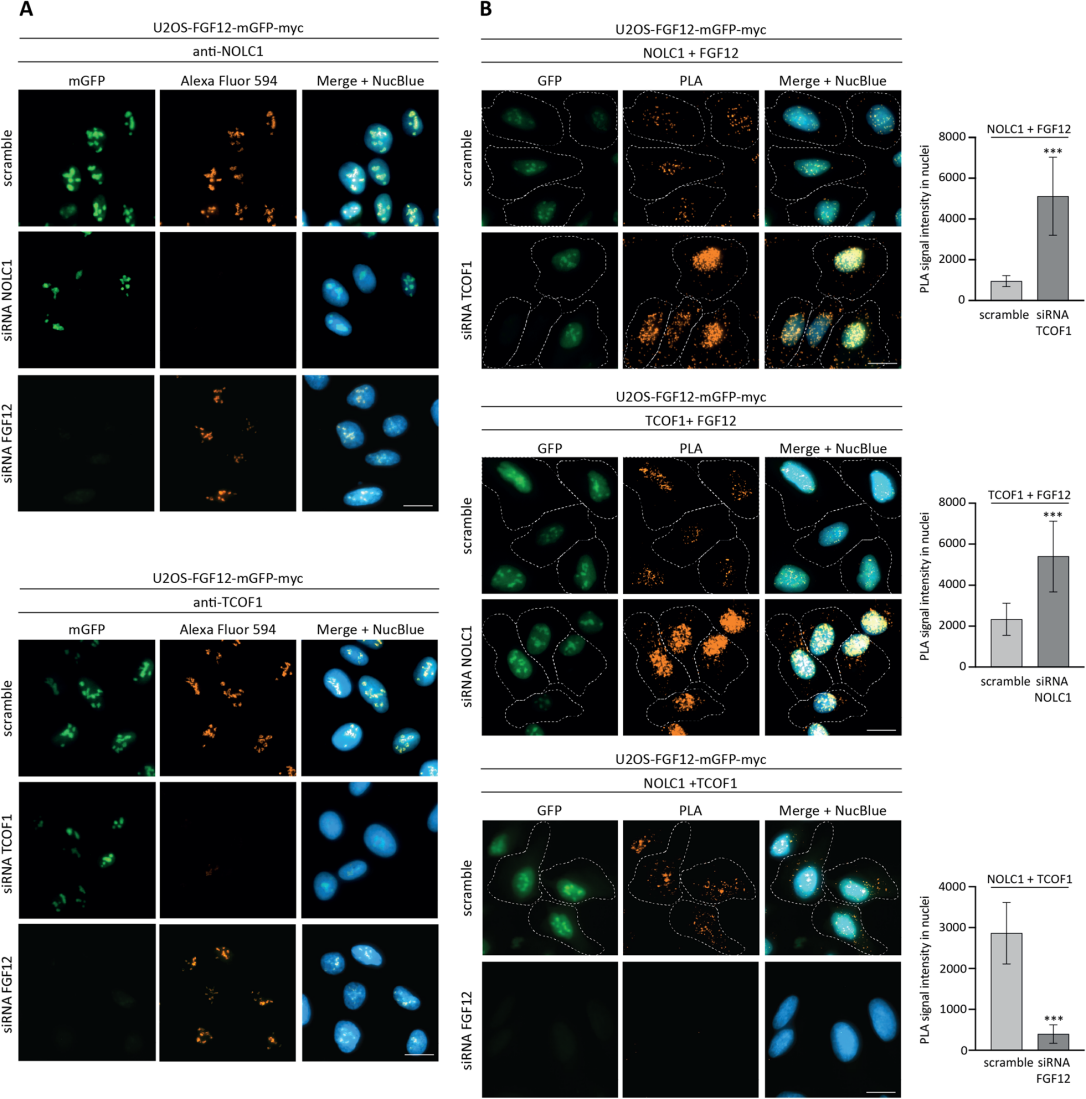
Role of FGF12 in the nucleolar localization of NOLC1 and TCOF1 and the assembly of their complexes. **A** Fluorescence microscopy analysis of the nucleolar localization of FGF12, NOLC1 and TCOF1 upon silencing of individual proteins. U2OS-FGF12-mGFP-myc cells, transfected with siRNA targeting NOLC1, TCOF1, FGF12 or scramble siRNA (control), were fixed, permeabilizated and stained with anti-NOLC1 or anti-TCOF1 primary antibodies and Alexa Fluor 594-conjugated secondary antibody. Cell nuclei were labeled with NucBlue Live and cells were analyzed by fluorescence microscopy. The scale bar represents 20 μm. **B** Fluorescence images of in situ PLA using anti-FGF12, anti-NOLC1 and anti-TCOF1 antibodies in U2OS-FGF12-mGFP-myc cells upon NOLC1, TCOF1 (n = 3) or FGF2 (n = 2) silencing. Cell nuclei were labeled with NucBlue Live and cells were analyzed by fluorescence microscopy. The dashed line indicates the cell area. The scale bar represents 20 μm. Data shown in the graphs are PLA signal intensity in nuclei. Student’s t-test was applied for statistical analysis; ***p < 0.001

To study the importance of NOLC1 and TCOF1 for the nucleolar localization of FGF12, we knocked-down NOLC1 or TCOF1 with siRNA in U2OS-FGF12-mGFP-myc cells (Fig. 4A, Additional file 6: Fig. S5). Silencing of either NOLC1 or TCOF1 had no effect on the nucleolar localization of FGF12-mGFP-myc (Fig. 4A). Furthermore, FGF12 knock-down also did not affect the localization of NOCL1 and TCOF1.

However, PLA experiments revealed that silencing of only one binding partner, NOLC1 or TCOF1, resulted in a partial re-localization of FGF12/TCOF1 and FGF12/NOLC1 complexes from the nucleolus to the nucleus (Fig. 4B). In addition, PLA signals for FGF12/TCOF1 and FGF12/NOLC1 pairs were significantly enhanced upon NOLC1 and TCOF1 knocking-down (Fig. 4B). A similar effect was observed in HEK293 cells (Additional file 7: Fig. S6). These data indicate that the presence of NOLC1 or TCOF1 is not required for nucleolar localization of FGF12. However, silencing of one of the FGF12 binding proteins (NOLC1 or TCOF1) resulted in increased level of FGF12 in complex with the other partner.

Surprisingly, FGF12 silencing resulted in complete inhibition of the interaction between NOLC1 and TCOF1, demonstrating that FGF12 is an integral component of NOLC1/TCOF1 complexes (Fig. 4B).

## Discussion

FHFs are pleiotropic proteins whose dysregulation is associated with disorders of the nervous system, cardiac diseases and cancer [19, 39–53]. The specificity of FHFs function appears to be largely determined by their subcellular localization. FHF members lack a signal sequence for secretion and have therefore been recognized as intracellular proteins, acting mainly as regulators of the plasma membrane-embedded voltage-gated ion channels [30, 54–58]. FHFs have been identified in the cytosol and in the nucleus. Although evidence for FHFs secretion is still lacking, several studies indicate that exogenous FHFs bind and activate FGFRs, triggering a protective response of the cell by preventing apoptosis [9, 33–35].

In this study, we have revealed a novel and very intriguing localization of FHF proteins. We found that all FHF family members reside in the nucleolus, however we observed that subcellular distribution (into nucleolus, nucleus and cytosol) differs between distinct FHF members. Furthermore, using FGF12 as an exemplary FHF, we performed the first MS-based identification of FHF cellular interaction network. Among the identified binding partners of FGF12 we found several nucleolar proteins, including NOLC1 and TCOF1. NOLC1 is a cellular target of the anti-cancer drug doxorubicin and natively unfolded scaffold protein that shuttles between the nucleolus and the cytosol, affecting the cellular localization of its binding partners [59–61]. In the nucleolus, NOLC1 forms a complex with its paralogue TCOF1 [62], a protein mutated in ribosomopathy manifested as Treacher-Collins Syndrome (TCS). Assembly of the NOLC1/TCOF1 complex occurs in a ubiquitination-dependent manner, and this complex links RNA polymerase I to ribosome-modifying enzymes, to regulate the translation process of cells differentiating towards neural crest specification [62–64]. NOLC1 and TCOF1 are also involved in maintaining genomic stability by acting as DNA damage response (DDR) factors [65].

Here, we have provided robust evidence for the unexpected involvement of FGF12 in nucleolar NOLC1/TCOF1 complexes. Our data indicate that nucleolar localization of FGF12 is independent of the presence of NOLC1 or TCOF1. However, both proteins are required to form FGF12 nucleolar complexes, as knock-down of NOLC1 or TCOF1 partially relocates FGF12/TCOF1 and FGF12/NOLC1 complexes to the nucleus. Silencing one of the FGF12 binders (NOLC1 or TCOF1) increases the level of the FGF12 complex with the other partner. Moreover, FGF12 appears to be central to the NOLC1/TCOF1 interaction. The binding of FGF12 to NOLC1 and TCOF1 is phosphorylation-dependent. Since NOLC1, TCOF1 and FHF are CK2 substrates or CK2 binding partners [65, 66], CK2 may constitute a major regulator for nucleolar assembly of the FGF12/NOLC1/TCOF1 complex.

At this stage, the role of nucleolar FGF12/NOLC1/TCOF1 complex is still unclear, but it is tempting to speculate that FGF12 may be an important player either in ribosome biogenesis or the DDR. While nucleolar localization and interaction with NOLC1 are conserved for all members of the FHF family, only FGF12 binds both NOLC1 and TCOF1. These findings suggest that distinct FHF proteins may contribute differently to NOLC1/TCOF1 activities. As FHFs and NOLC1/TCOF1 are implicated in severe disease, including epilepsy, cancer and ribosomopathy, future studies should aim to decipher the functional interplay between FHF proteins and NOLC1/TCOF1.

## Supplementary Information

**Additional file 1: Table S1.** Results of MS-based peptide identification for proteins that bind specifically to FGF12-His in pull down experiments. ID is the Uniprot protein identifier. The score and peptide match for each of two repeats of the experiment are given.

**Additional file 2: Fig. S1.** Figure S1. Cellular localization of FHF proteins. **A** Western blotting analysis of whole cell lysates detecting mGFP in U2OS cells stably transfected with FGF11-mGFP-myc, FGF12-mGFP-myc, FGF13-mGFP-myc, FGF14-mGFP-myc or U2OS cells (control) to confirm the expression of fusion proteins. **B** Localization of FHF proteins in U2OS cells stably transfected with FGF11-mGFP-myc, FGF12-mGFP-myc, FGF13-mGFP-myc and FGF14-mGFP-myc. Nuclei were labeled with NucBlue Live and cells were analyzed with fluorescence microscopy. The dashed line indicates the cell area. The scale bar represents 20 μm. **C** Quantification of the amount of fluorescent proteins in each compartment (cytoplasm, nucleus, nucleolus) including mean fluorescence intensity and compartment area. Data presented are means ± SD of 20 cells. Student's t-test was used for statistical analysis; ***p < 0.001; **p < 0.01. **D** U2OS-FGF11-mGFP-myc, U2OS-FGF12-mGFP-myc, U2OS-FGF13-mGFP-myc and U2OS-FGF14-mGFP-myc cells were washed, lysed and fractionated into cytoplasmic and nuclear fractions. FHFs-mGFP-myc were extracted from each fraction by adsorption onto anti-myc resin, separated by SDS-PAGE and analyzed by western blotting. ERK1/2 and histone H3 served as cytoplasmic and nuclear marker proteins, respectively.

**Additional file 3: Fig. S2.** Streptavidin agarose pull-down from U2OS cells stably expressing FGF12-SBP. **A** Western blotting of cell lysates of U2OS-FGF12-SBP and U2OS cells (control) to confirm FGF12-SBP expression. **B** U2OS-FGF12-SBP and U2OS cells were lysed and co-purification of NOLC1 and TCOF1 with FGF12-SBP on streptavidin agarose was verified by SDS-PAGE and western blotting. **C** Streptavidin-agarose pull-down from U2OS-FGF12-SBP cells to purify FGF12-SBP complexes under native conditions. Bound proteins were eluted with biotin and separated by 2D-BN-PAGE. Co-migration of FGF12-SBP and NOLC1 or TCOF1 was determined by western blotting.

**Additional file 4: Fig. S3.** Negative controls for in situ PLA performed in U2OS-FGF12-mGFP-myc cells. PLA in U2OS-FGF12-mGFP-myc cells treated with a single antibody or in untreated cells. Nuclei were labeled with NucBlue Live and cells were analyzed with fluorescence microscopy. Scale bar represents 20 μm.

**Additional file 5: Fig. S4.** PLA in HEK 293 cells lacking ectopic FGF12. Fluorescence images of in situ PLA using rabbit anti-FGF12 and mouse anti-NOLC1 or anti-TCOF1 antibody in HEK 293 cells. HEK 293 cells treated with a single antibody or untreated showed no signal and served as negative controls. Nuclei were labeled with NucBlue Live and cells were analyzed with fluorescence microscopy. The dashed line indicates the cell area. The scale bar represents 20 μm.

**Additional file 6: Fig. S5.** Efficiency of NOLC1, TCOF1 and FGF12 knock-down in U2OS-FGF12-mGFP-myc cells. Western blotting analysis of cell lysates of U2OS-FGF12-mGFP-myc cells treated with siRNA against NOLC1, TCOF1 or FGF12.

**Additional file 7: Fig. S6.** Effect of NOLC1 and TCOF1 knock-down on the nucleolar localization of FGF12 and its interaction with NOLC1 and TCOF1. HEK 293 cells were transfected with NOLC1/TCOF1-targeting siRNA or scramble siRNA (control). Fluorescence images of in situ PLA using a mixture of rabbit anti-FGF12 and mouse anti-NOLC1 or anti-TCOF1 antibodies. Cell nuclei were labeled with NucBlue Live and cells were analyzed with fluorescence microscopy. The dashed line indicates the cell area. The scale bar represents 20 μm.

## Data Availability

The datasets used in this study are available from the corresponding author on reasonable request.

## Abbreviations

BCLAF1: Bcl-2-associated transcription factor 1
CBB: Coomassie brilliant blue
CIP: Calf intestinal alkaline phosphatase
CK2: Casein kinase 2
DDR: DNA damage response
ERK1/2: Extracellular signal-regulated kinases 1 and 2
FGFR: Fibroblast growth factor receptor
FGF: Fibroblast growth factor
FHF: Fibroblast homologous factor
GFP: Green fluorescent protein
MS: Mass spectrometry
NOLC1: Nucleolar and coiled-body phosphoprotein 1
PLA: Proximity ligation assay
RSL1D1: Ribosomal L1 domain-containing protein 1
RPS14: Ribosomal protein S14
RPL18A: Ribosomal protein L18
SBP: Streptavidin binding peptide
TCOF1: Treacle ribosome biogenesis factor 1
TCS: Treacher Collins syndrome
THRAP3: Thyroid hormone receptor-associated protein 3
