# Supplementary material for: FGF12 is a novel component of the nucleolar NOLC1/TCOF1 ribosome biogenesis complex
Source: Cell Commun Signal. 2022 Nov 21;20:182. doi: 10.1186/s12964-022-01000-4 (PMC9677703; doi:10.1186/s12964-022-01000-4)
Supplement: Supplementary file 2 — Additional file 1: Table S1. Results of MS-based peptide identification for proteins that bind specifically to FGF12-His in pull down experiments. ID is the Uniprot protein identifier. The score and peptide match for each of two repeats of the experiment are given. [file 12964_2022_1000_MOESM2_ESM.docx]

**Supplementary Figure Legends**

**Figure S1. Cellular localization of FHF proteins.**

**A.** Western blotting analysis of whole cell lysates detecting mGFP in U2OS cells stably transfected with FGF11-mGFP-myc, FGF12-mGFP-myc, FGF13-mGFP-myc, FGF14-mGFP-myc or U2OS cells (control) to confirm the expression of fusion proteins. **B.** Localization of FHF proteins in U2OS cells stably transfected with FGF11-mGFP-myc, FGF12-mGFP-myc, FGF13-mGFP-myc and FGF14-mGFP-myc. Nuclei were labeled with NucBlue Live and cells were analyzed with fluorescence microscopy. The dashed line indicates the cell area. The scale bar represents 20 μm. **C.** Quantification of the amount of fluorescent proteins in each compartment (cytoplasm, nucleus, nucleolus) including mean fluorescence intensity and compartment area. Data presented are means ± SD of 20 cells. Student's t-test was used for statistical analysis; *** p < 0.001; ** p < 0.01 . **D.** U2OS-FGF11-mGFP-myc, U2OS-FGF12-mGFP-myc, U2OS-FGF13-mGFP-myc and U2OS-FGF14-mGFP-myc cells were washed, lysed and fractionated into cytoplasmic and nuclear fractions. FHFs-mGFP-myc were extracted from each fraction by adsorption onto anti-myc resin, separated by SDS-PAGE and analyzed by western blotting. ERK1/2 and histone H3 served as cytoplasmic and nuclear marker proteins, respectively.

**Figure S2. Streptavidin agarose pull-down from U2OS cells stably expressing FGF12-SBP.**

**A.** Western blotting of cell lysates of U2OS-FGF12-SBP and U2OS cells (control) to confirm FGF12-SBP expression. **B.** U2OS-FGF12-SBP and U2OS cells were lysed and co-purification of NOLC1 and TCOF1 with FGF12-SBP on streptavidin agarose was verified by SDS-PAGE and western blotting. **C.** Streptavidin-agarose pull-down from U2OS-FGF12-SBP cells to purify FGF12-SBP complexes under native conditions. Bound proteins were eluted with biotin and separated by 2D-BN-PAGE. Co-migration of FGF12-SBP and NOLC1 or TCOF1 was determined by western blotting.

**Figure S3. Negative controls for *in situ*** **PLA performed in U2OS-FGF12-mGFP-myc cells.**

PLA in U2OS-FGF12-mGFP-myc cells treated with a single antibody or in untreated cells. Nuclei were labeled with NucBlue Live and cells were analyzed with fluorescence microscopy. Scale bar represents 20 μm.

**Figure S4.** **PLA in HEK 293 cells lacking ectopic FGF12**.

Fluorescence images of *in situ* PLA using rabbit anti-FGF12 and mouse anti-NOLC1 or anti-TCOF1 antibody in HEK 293 cells. HEK 293 cells treated with a single antibody or untreated showed no signal and served as negative controls. Nuclei were labeled with NucBlue Live and cells were analyzed with fluorescence microscopy. The dashed line indicates the cell area. The scale bar represents 20 μm.

**Figure S5. Efficiency of NOLC1, TCOF1 and FGF12 knock-down in U2OS-FGF12-mGFP-myc cells.**

Western blotting analysis of cell lysates of U2OS-FGF12-mGFP-myc cells treated with siRNA against NOLC1, TCOF1 or FGF12.

**Figure S6. Effect of NOLC1 and TCOF1 knock-down on the nucleolar localization of FGF12 and its interaction with NOLC1 and TCOF1.**

HEK 293 cells were transfected with NOLC1/TCOF1-targeting siRNA or scramble siRNA (control). Fluorescence images of *in situ* PLA using a mixture of rabbit anti-FGF12 and mouse anti-NOLC1 or anti-TCOF1 antibodies. Cell nuclei were labeled with NucBlue Live and cells were analyzed with fluorescence microscopy. The dashed line indicates the cell area. The scale bar represents 20 μm.

**Table S1. Results of MS-based peptide identification for proteins that bind specifically to FGF12-His in pull down experiments.** ID is the Uniprot protein identifier. The score and peptide match for each of two repeats of the experiment are given.

| Position | ID | Protein name | Score  (peptide matches) |
| --- | --- | --- | --- |
| 1 | **Q14978** | **Nucleolar and coiled-body phosphoprotein 1** | 1433.0 (21)  1137.0 (23) |
| 2 | P68371 | Tubulin beta-4B chain | 1065.0 (16)  455.0 (9) |
| 3 | Q9UQ35 | Serine/arginine repetitive matrix protein 2 | 1649.0 (39)  828.0 (20) |
| 4 | Q9NYF8 | Bcl-2-associated transcription factor 1 | 1830.0 (31)  1447.0 (31) |
| 5 | Q9Y2W1 | Thyroid hormone receptor-associated protein 3 | 1560.0 (18)  983.0 (16) |
| 6 | P67809 | Y-box-binding protein 1 | 1350.0 (7)  1017.0 (14) |
| 7 | Q9H307 | Pinin | 619.0 (14)  334.0 (7) |
| 8 | Q02878 | 60S ribosomal protein L6 | 274.0 (4)  584.0 (8) |
| 9 | P26373 | 60S ribosomal protein L13 | 1424.0 (12)  435.0 (9) |
| 10 | **Q13428** | **Treacle protein** | 648.0 (17)  562.0 (17) |
| 11 | O76021 | Ribosomal L1 domain-containing protein 1 | 140.0 (4)  549.0 (15) |
| 12 | P35579 | Myosin-9 | 540.0 (15)  339.0 (7) |
| 13 | P62701 | 40S ribosomal protein S4, X isoform | 477.0 (10)  507.0 (10) |
| 14 | P16104 | Histone H2AX | 270.0 (4)  216.0 (3) |
| 15 | Q92522 | Histone H1x | 441.0 (4)  389.0 (6) |
| 16 | P18124 | 60S ribosomal protein L7 | 325.0 (2)  269.0 (7) |
| 17 | Q8IYB3 | Serine/arginine repetitive matrix protein 1 | 142.0 (4)  376.0 (10) |
| 18 | P62753 | 40S ribosomal protein S6 | 535.0 (3)  417.0 (8) |
| 19 | Q07020 | 60S ribosomal protein L18 | 205.0 (3)  335.0 (4) |
| 20 | Q00839 | Heterogeneous nuclear ribonucleoprotein U | 268.0 (3)  481.0 (10) |
| 21 | P46781 | 40S ribosomal protein S9 | 324.0 (2)  175.0 (4) |
| 22 | P07910 | Heterogeneous nuclear ribonucleoproteins C1/C2 | 321.0 (4)  416.0 (5) |
| 23 | P62917 | 60S ribosomal protein L8 | 336.0 (3)  260.0 (5) |
| 24 | P62750 | 60S ribosomal protein L23a | 211.0 (5)  485.0 (8) |
| 25 | P62263 | 40S ribosomal protein S14 | 255.0 (4)  197.0 (4) |
| 26 | P62424 | 60S ribosomal protein L7a | 287.0 (6)  290.0 (6) |
| 27 | P36578 | 60S ribosomal protein L4 | 260.0 (3)  187.0 (6) |
| 28 | P61254 | 60S ribosomal protein L26 | 246.0 (5)  199.0 (4) |
| 29 | P84098 | 60S ribosomal protein L19 | 496.0 (4)  169.0 (3) |
| 30 | P15880 | 40S ribosomal protein S2 | 336.0 (6)  100.0 (3) |
| 31 | Q86VM9 | Zinc finger CCCH domain-containing protein 18 | 271.0 (3)  155.0 (3) |
| 32 | Q9NR30 | Nucleolar RNA helicase 2 | 94.0 (2)  330.0 (9) |
| 33 | P19338 | Nucleolin | 312.0 (5)  190.0 (6) |
| 34 | P62266 | 40S ribosomal protein S23 | 283.0 (2)  281.0 (4) |
| 35 | O00422 | Histone deacetylase complex subunit SAP18 | 188.0 (2)  218.0 (5) |
| 36 | P62277 | 40S ribosomal protein S13 | 144.0 (3)  238.0 (5) |
| 37 | P06748 | Nucleophosmin | 256.0 (3)  120.0 (2) |
| 38 | P68431 | Histone H3.1 | 252.0 (6)  133.0 (4) |
| 39 | Q12797 | Aspartyl/asparaginyl beta-hydroxylase | 170.0 (2)  190.0 (2) |
| 40 | P61247 | 40S ribosomal protein S3a | 140.0 (4)  289.0 (6) |
| 41 | P46776 | 60S ribosomal protein L27a | 128.0 (2)  273.0 (4) |
| 42 | Q9P2E9 | Ribosome-binding protein 1 | 80.0 (3)  252.0 (6) |
| 43 | P62241 | 40S ribosomal protein S8 | 182.0 (3)  141.0 (3) |
| 44 | P18621 | 60S ribosomal protein L17 | 274.0 (3)  178.0 (3) |
| 45 | P16989 | Y-box-binding protein 3 | 130.0 (2)  168.0 (3) |
| 46 | P21333 | Filamin-A | 119.0 (4)  244.0 (3) |
| 47 | P68104 | Elongation factor 1-alpha 1 | 273.0 (4)  75.0 (2) |
| 48 | Q15149 | Plectin | 177.0 (5)  173.0 (5) |
| 49 | O43390 | Heterogeneous nuclear ribonucleoprotein R | 101.0 (2)  187.0 (2) |
| 50 | Q01130 | Serine/arginine-rich splicing factor 2 | 240.0 (3)  129.0 (2) |
| 51 | P39023 | 60S ribosomal protein L3 | 157.0 (2)  162.0 (4) |
| 52 | Q08170 | Serine/arginine-rich splicing factor 4 | 130.0 (3)  135.0 (3) |
| 53 | P84090 | Enhancer of rudimentary homolog | 130.0 (3)  125.0 (2) |
| 54 | P62280 | 40S ribosomal protein S11 | 200.0 (4)  157.0 (3) |
| 55 | Q07955 | Serine/arginine-rich splicing factor 1 | 305.0 (8)  57.0 (2) |
| 56 | P39019 | 40S ribosomal protein S19 | 84.0 (3)  71.0 (2) |
| 57 | P62995 | Transformer-2 protein homolog beta | 116.0 (3)  125.0 (4) |
| 58 | Q13595 | Transformer-2 protein homolog alpha | 133.0 (5)  84.0 (3) |
| 59 | Q13247 | Serine/arginine-rich splicing factor 6 | 179.0 (5)  104.0 (3) |
| 60 | P62081 | 40S ribosomal protein S7 | 160.0 (2)  49.0 (2) |
| 61 | P46013 | Proliferation marker protein Ki-67 | 84.0 (2)  123.0 (4) |
| 62 | P61978 | Heterogeneous nuclear ribonucleoprotein K | 146.0 (3)  60.0 (2) |
| 63 | Q14966 | Zinc finger protein 638 | 72.0 (2)  130.0 (3) |
| 64 | P84103 | Serine/arginine-rich splicing factor 3 | 129.0 (3)  84.0 (2) |
| 65 | P62906 | 60S ribosomal protein L10a | 59.0 (2)  126.0 (3) |
| 66 | Q02543 | 60S ribosomal protein L18a | 89.0 (2)  89.0 (3) |
| 67 | P11142 | Heat shock cognate 71 kDa protein | 99.0 (4)  110.0 (3) |
| 68 | P46087 | Probable 28S rRNA (cytosine(4447)-C(5))-methyltransferase | 81.0 (2)  86.0 (3) |
| 69 | P49207 | 60S ribosomal protein L34 | 98.0 (2)  64.0 (2) |
| 70 | Q9NVP1 | ATP-dependent RNA helicase DDX18 | 63.0 (2)  93.0 (2) |
| 71 | P17844 | Probable ATP-dependent RNA helicase DDX5 | 87.0 (3)  87.0 (3) |
| 72 | Q92841 | Probable ATP-dependent RNA helicase DDX17 | 81.0 (3)  64.0 (2) |
| 73 | Q96GQ7 | Probable ATP-dependent RNA helicase DDX27 | 62.0 (2)  65.0 (2) |
